# A self-supervised learning strategy for postoperative brain cavity segmentation simulating resections

**DOI:** 10.1007/s11548-021-02420-2

**Published:** 2021-06-13

**Authors:** Fernando Pérez-García, Reuben Dorent, Michele Rizzi, Francesco Cardinale, Valerio Frazzini, Vincent Navarro, Caroline Essert, Irène Ollivier, Tom Vercauteren, Rachel Sparks, John S. Duncan, Sébastien Ourselin

**Affiliations:** 1 Department of Medical Physics and Biomedical Engineering, UCL, London, UK; 2 Wellcome/EPSRC Centre for Interventional and Surgical Sciences, UCL, London, UK; 3 School of Biomedical Engineering & Imaging Sciences, King’s College London, London, UK; 4 “C. Munari” Epilepsy Surgery Centre ASST GOM Niguarda, Milan, Italy; 5 Paris Brain Institute, ICM, INSERM, CNRS, 75013 Paris, France; 6 Sorbonne Université, 75013 Paris, France; 7 Epilepsy Unit, Reference Center for Rare Epilepsies, and Departement of Clinical Neurophysiology, AP-HP, Pitié-Salpêtrière Hospital, 75013 Paris, France; 8 ICube, Université de Strasbourg, CNRS (UMR 7357), Strasbourg, France; 9 Department of Neurosurgery, Strasbourg University Hospital, Strasbourg, France; 10 UCL Queen Square Institute of Neurology, London, UK; 11 National Hospital for Neurology and Neurosurgery, London, UK

**Keywords:** Resective neurosurgery, Cavity segmentation, Lesion simulation, Self-supervised learning, Neuroimaging

## Abstract

**Purpose:**

Accurate segmentation of brain resection cavities (RCs) aids in postoperative analysis and determining follow-up treatment. Convolutional neural networks (CNNs) are the state-of-the-art image segmentation technique, but require large annotated datasets for training. Annotation of 3D medical images is time-consuming, requires highly trained raters and may suffer from high inter-rater variability. Self-supervised learning strategies can leverage unlabeled data for training.

**Methods:**

We developed an algorithm to simulate resections from preoperative magnetic resonance images (MRIs). We performed self-supervised training of a 3D CNN for RC segmentation using our simulation method. We curated EPISURG, a dataset comprising 430 postoperative and 268 preoperative MRIs from 430 refractory epilepsy patients who underwent resective neurosurgery. We fine-tuned our model on three small annotated datasets from different institutions and on the annotated images in EPISURG, comprising 20, 33, 19 and 133 subjects.

**Results:**

The model trained on data with simulated resections obtained median (interquartile range) Dice score coefficients (DSCs) of 81.7 (16.4), 82.4 (36.4), 74.9 (24.2) and 80.5 (18.7) for each of the four datasets. After fine-tuning, DSCs were 89.2 (13.3), 84.1 (19.8), 80.2 (20.1) and 85.2 (10.8). For comparison, inter-rater agreement between human annotators from our previous study was 84.0 (9.9).

**Conclusion:**

We present a self-supervised learning strategy for 3D CNNs using simulated RCs to accurately segment real RCs on postoperative MRI. Our method generalizes well to data from different institutions, pathologies and modalities. Source code, segmentation models and the EPISURG dataset are available at https://github.com/fepegar/resseg-ijcars.

## Introduction

### Motivation

Approximately one-third of epilepsy patients are drug-resistant. If the epileptogenic zone (EZ), i.e., “the area of cortex indispensable for the generation of clinical seizures” [26], can be localized, resective surgery to remove the EZ may be curative. Currently, 40% to 70% of patients with refractory focal epilepsy are seizure-free after surgery [16]. This is, in part, due to limitations identifying the EZ. Retrospective studies relating presurgical clinical features and resected brain structures to surgical outcome provide useful insight to guide EZ resection [16]. To quantify resected structures, first, the resection cavity (RC) must be segmented on the postoperative magnetic resonance image (MRI). A preoperative image with a corresponding brain parcellation can then be registered to the postoperative MRI to identify resected structures.

RC segmentation is also necessary in other applications. For neuro-oncology, the gross tumor volume, which is the sum of the RC and residual and residual tumor volumes, is estimated for postoperative radiotherapy [10].

Despite recent efforts to segment RCs in the context of brain cancer [10, 18], little research has been published in the context of epilepsy surgery. Furthermore, previous work is limited by the lack of benchmark datasets, released code or trained models, and evaluation is restricted to single-institution datasets used for both training and testing.

### Related works

After surgery, RCs fill with cerebrospinal fluid (CSF). This causes an inherent uncertainty in delineating RCs adjacent to structures such as sulci, ventricles or edemas. Nonlinear registration has been presented to segment the RC for epilepsy [6] and brain tumor [4] surgeries by detecting non-corresponding regions between pre- and postoperative images. However, evaluation of these methods was restricted to a very small number of images. Furthermore, in cases with intensity changes due to the resection (e.g., brain shift, atrophy, fluid filling), non-corresponding voxels may not correspond to the RC.

Decision forests were presented for brain cavity segmentation after glioblastoma surgery, using four MRI modalities [18]. These methods, which aggregate hand-crafted features extracted from all modalities to train a classifier, can be sensitive to signal inhomogeneity and unable to distinguish regions with intensity patterns similar to CSF from RCs. Recently, a 2D convolutional neural network (CNN) was trained to segment the RC on MRI slices in 30 glioblastoma patients [10]. They obtained a ‘median (interquartile range)’ Dice score coefficient (DSC) of 84 (10) compared to ground-truth labels by averaging predictions across anatomical axes to compute the 3D segmentation. While these approaches require four modalities to segment the resection cavity, some of the modalities are often unavailable in clinical settings [9]. Furthermore, code and datasets are not publicly available, hindering a fair comparison across methods. Applying these techniques requires curating a dataset with manually obtained annotations to train the models, which is expensive.

Unsupervised learning methods can leverage large, unlabeled medical image datasets during training. In self-supervised learning, training instances are generated automatically from unlabeled data and used to train a model to perform a pretext task. The model can be fine-tuned on a smaller labeled dataset to perform a downstream task [5]. The pretext and downstream tasks may be the same. For example, a CNN was trained to reconstruct a skull bone flap by simulating craniectomies on CT scans [17]. Lesions simulated in chest CT of healthy subjects were used to train models for nodule detection, improving accuracy compared to training on a smaller dataset of real lesions [25].

### Contributions

We present a self-supervised learning approach to train a 3D CNN to segment brain RCs from $T_{1}$-weighted ($T_{1}$w) MRI without annotated data, by simulating resections during training. We ensure our work is reproducible by releasing the source code for resection simulation and CNN training, the trained CNN and the evaluation dataset. To the best of our knowledge, we introduce the first open annotated dataset of postoperative MRI for epilepsy surgery.

This work extends our conference paper [22] as follows: (1) we performed a more comprehensive evaluation, assessing the effect of the resection simulation shape on performance and evaluating datasets from different institutions and pathologies; (2) we formalized our transfer learning strategy.

## Methods

### Learning strategy

#### Problem statement

The overall objective is to automatically segment RCs from postoperative $T_{1}$w MRI using a CNN $f_{\varvec{\theta }}$ parameterized by weights $\varvec{\theta }$. Let $\varvec{X}_{\text {post}} : \varOmega \rightarrow {\mathbb {R}}$ and $\varvec{Y}_{\text {cavity}}: \varOmega \rightarrow \{ 0, 1 \}$ be a postoperative $T_{1}$w MRI and its cavity segmentation label, respectively, where $\varOmega \subset {\mathbb {R}}^3$. $\varvec{X}_{\text {post}}$ and $\varvec{Y}_{\text {cavity}}$ are drawn from the data distribution ${\mathcal {D}}_{\text {post}}$. In model training, the aim is to minimize the expected discrepancy between the label $\varvec{Y}_{\text {cavity}}$ and network prediction $f_{\varvec{\theta }}(\varvec{X}_{\text {post}})$. Let ${\mathcal {L}}$ be a loss function that estimates this discrepancy (e.g., Dice loss). The optimization problem for the network parameters $\varvec{\theta }$ is:

1 $$\begin{aligned} \varvec{\theta }^* = {{\,\mathrm{arg\,min}\,}}_{\varvec{\theta }} {\mathbb {E}}_{{\mathcal {D}}_{\text {post}}}\left[ {\mathcal {L}}\left( f_{\varvec{\theta }} \left( \varvec{X}_{\text {post}}\right) , \varvec{Y}_{\text {cavity}}\right) \right] \end{aligned}$$

In a fully supervised setting, a labeled dataset $D_{\text {post}}= \{ (\varvec{X}_{\text {post}_i}, \varvec{Y}_{\text {cavity}_i}) \}_{i = 1}^{n_{\text {post}}}$ is employed to estimate the expectation defined in (1) as:

2 $$\begin{aligned}&{\mathbb {E}}_{{\mathcal {D}}_{\text {post}}}\left[ {\mathcal {L}}\left( f_{\varvec{\theta }} \left( \varvec{X}_{\text {post}}\right) , \varvec{Y}_{\text {cavity}}\right) \right] \nonumber \\&\quad \approx \frac{1}{n_{\text {post}}} \sum _{i=1}^{n_{\text {post}}} {\mathcal {L}}(f_{\varvec{\theta }}(\varvec{X}_{\text {post}_i}), \varvec{Y}_{\text {post}_i}) \end{aligned}$$

In practice, CNNs typically require an annotated dataset with a large $n_{\text {post}}$ to generalize well for unseen instances. However, given the time and expertise required to annotate scans, $n_{\text {post}}$ is often small. We present a method to artificially increase $n_{\text {post}}$ by simulating postoperative MRIs and associated labels from preoperative scans.

#### Simulation for domain adaptation and self-supervised learning

Let $D_{\text {pre}}= \{ \varvec{X}_{\text {pre}_i} \}_{i = 1}^{n_{\text {pre}}}$ be a dataset of preoperative $T_{1}$w MRI, drawn from the data distribution ${\mathcal {D}}_{\text {pre}}$. We propose to generate a simulated postoperative dataset $D_{\text {sim}}= \{ (\varvec{X}_{\text {sim}_i}, \varvec{Y}_{\text {sim}_i}) \}_{i = 1}^{n_{\text {sim}}}$ using the preoperative dataset $D_{\text {pre}}$. Specifically, we aim to build a generative model $\phi _{\text {sim}}: \varvec{X}_{\text {pre}}\mapsto (\varvec{X}_{\text {sim}}, \varvec{Y}_{\text {sim}})$ that transforms preoperative images into simulated, annotated postoperative images that imitate instances drawn from the postoperative data distribution ${\mathcal {D}}_{\text {post}}$. $D_{\text {sim}}$ can then be used to estimate the expectation in (1):

3 $$\begin{aligned}&{\mathbb {E}}_{{\mathcal {D}}_{\text {post}}}\left[ \ {\mathcal {L}}\left( f_{\varvec{\theta }} \left( \varvec{X}_{\text {post}}\right) , \varvec{Y}_{\text {cavity}}\right) \right] \nonumber \\&\quad \approx \frac{1}{n_{\text {sim}}}\sum _{i=1}^{n_{\text {sim}}} {\mathcal {L}}(f_{\varvec{\theta }}(\varvec{X}_{\text {sim}_i}), \varvec{Y}_{\text {sim}_i}) \end{aligned}$$

Simulated images can be generated from any unlabeled preoperative dataset. Therefore, the size of the simulated dataset can be much greater than the annotated dataset $D_{\text {post}}$, i.e., $n_{\text {sim}}\gg n_{\text {post}}$. The network parameters $\varvec{\theta }$ can be optimized by minimizing (3) using stochastic gradient descent, leading to a trained predictive function $f_{\varvec{\theta }_{\text {sim}}}$. Finally, $f_{\varvec{\theta }_{\text {sim}}}$ can be fine-tuned on $D_{\text {post}}$ to improve performance on the postoperative domain ${\mathcal {D}}_{\text {post}}$.

### Resection simulation for self-supervised learning

$\phi _{\text {sim}}$ takes images from ${\mathcal {D}}_{\text {pre}}$ to generate training instances by simulating a realistic shape, location and intensity pattern for the RC. We present simulation of cavity shape and label in sections “Initial cavity shape” and “Cavity label”, respectively. In section “Simulating cavities filled with CSF”, we present our method to generate the resected image.

#### Initial cavity shape

To simulate a realistic RC, we consider its topological and geometric properties: it is a single volume with a non-smooth boundary. We generate a geodesic polyhedron with frequency *f* by subdividing the edges of an icosahedron *f* times and projecting each vertex onto a parametric sphere with a unit radius centered at the origin. This polyhedron models a spherical surface $S = \{ V, F \}$ with vertices $V = \left\{ \varvec{v}_i \in {\mathbb {R}}^3 \right\} _{i = 1}^{n_V}$ and faces $F = \left\{ \varvec{f}_k \in {\mathbb {N}}^3 \right\} _{k = 1}^{n_F}$, where $n_V$ and $n_F$ are the number of vertices and faces, respectively. Each face $\varvec{f}_k = \{ i_1^k, i_2^k, i_3^k \}$ is a sequence of three non-repeated vertex indices.

To create a non-smooth surface, *S* is perturbed with simplex noise [24], a procedural noise generated by interpolating pseudorandom gradients on a multidimensional simplicial grid. We chose simplex noise as it simulates natural-looking textures or terrains and is computationally efficient for multiple dimensions. The noise $\eta : {\mathbb {R}}^3 \rightarrow [-1, 1]$ at point $\varvec{p}\in {\mathbb {R}}^3$ is a weighted sum of the noise contribution for $\omega$ different octaves, with weights $\{\gamma ^ {n - 1}\}_{n = 1}^{\omega }$ controlled by the persistence parameter $\gamma$. The displacement $\delta$ of a vertex $\varvec{v}$ is:

4 $$\begin{aligned} \delta (\varvec{v}) = \eta \left( \frac{\varvec{v}+ \varvec{\mu } }{\zeta }, \omega , \gamma \right) \end{aligned}$$

where $\zeta$ is a scaling parameter to control smoothness and $\varvec{\mu }$ is a shifting parameter that adds stochasticity (equivalent to a random number generator seed). Each vertex $\varvec{v}_i$ is displaced radially to create a perturbed sphere: $V_{\delta } = \left\{ \varvec{v}_i + \delta (\varvec{v}_i) \frac{\varvec{v}_i}{\Vert \varvec{v}_i\Vert } \right\} _{i = 1}^{n_V} = \left\{ \varvec{v}_{\delta i} \right\} _{i = 1}^{n_V}$.

Next, a series of transforms is applied to $V_{\delta }$ to modify the mesh’s volume and shape. To add stochasticity, random rotations around each axis are applied to $V_{\delta }$ with the rotation transform $T_{\text {R}}(\varvec{\theta }_{\text {r}}) = R_x(\theta _x) \circ R_y(\theta _y) \circ R_z(\theta _z)$, where $\circ$ indicates a transform composition and $R_i(\theta _i)$ is a rotation of $\theta _i$ radians around axis *i*. $T_{\text {S}}(\varvec{r})$ is a scaling transform, where $(r_1, r_2, r_3) = \varvec{r}$ are semiaxes of an ellipsoid with volume *v* used to model the cavity shape. The semiaxes are computed as $r_1 = r$, $r_2 = \lambda r$ and $r_3 = r /\lambda$, where $r = (3 v / 4)^{1/3}$ and $\lambda$ controls the semiaxes length ratios.^1^ These transforms are applied to $V_{\delta }$ to define the initial resection cavity surface $S_{\text {E}} = \{ V_{\text {E}}, F \}$, where $V_{\text {E}} = \{ T_{\text {S}}(\varvec{r}) \circ T_{\text {R}}(\varvec{\theta }_{\text {r}})( \varvec{v}_{\delta i}) \} _{i = 1}^{n_V}$.

#### Cavity label

The simulated RC should not span both hemispheres or include extracerebral tissues such as bone or scalp. This section describes our method to ensure that the RC appears in anatomically plausible regions.

**Fig. 1:**
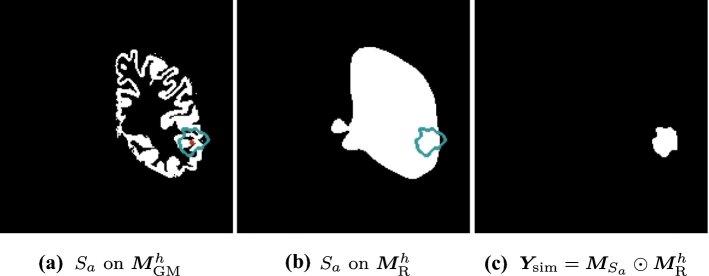
Simulation of the ground-truth cavity label. $S_a$ (blue) is computed by centering $S_{\text {E}}$ on $\varvec{a}$, a random positive voxel (red) of $\varvec{M}_{\text {GM}}^h$ (**a**). $\varvec{M}_{S_a}$ is a binary mask derived from $S_a$. $\varvec{Y}_{\text {sim}}$ (**c**) is the intersection of $\varvec{M}_{S_a}$ and $\varvec{M}_{\text {R}}^h$ (**b**)

A $T_{1}$w MRI is defined as $\varvec{X}_{\text {pre}}: \varOmega \rightarrow {\mathbb {R}}$. A full brain parcellation $\varvec{P} : \varOmega \rightarrow Z$ is generated [3] for $\varvec{X}_{\text {pre}}$, where *Z* is the set of segmented structures. A cortical gray matter mask $\varvec{M}_{\text {GM}}^h : \varOmega \rightarrow \{0, 1\}$ of hemisphere *h* is extracted from $\varvec{P}$, where *h* is randomly chosen from $H = \{\text {left}, \text {right}\}$ with equal probability.

A “resectable hemisphere mask” $\varvec{M}_{\text {R}}^h$ is generated from $\varvec{P}$ and *h* such that $\varvec{M}_{\text {R}}^h (\varvec{p}) = 1$ if ${\varvec{P}(\varvec{p}) \ne \{M_{\text {BG}}, M_{\text {BT}}, M_{\text {CB}}, M_{{\hat{h}}} \} }$ and 0 otherwise, where $M_{\text {BG}}$, $M_{\text {BT}}$, $M_{\text {CB}}$ and $M_{{\hat{h}}}$ are the labels in *Z* corresponding to the background, brainstem, cerebellum and contralateral hemisphere, respectively. $\varvec{M}_{\text {R}}^h$ is smoothed using a series of binary morphological operations, for realism.

A random voxel $\varvec{a} \in \varOmega$ is selected such that $\varvec{M}_{\text {GM}}^h(\varvec{a}) = 1$. A translation transform $T_{\text {T}}(\varvec{a} - \varvec{c})$ is applied to $S_{\text {E}}$, so $S_a = T_{\text {T}}(\varvec{a} - \varvec{c}) (S_{\text {E}})$ is centered on $\varvec{a}$.

A binary image $\varvec{M}_{S_a} : \varOmega \rightarrow \{ 0, 1 \}$ is generated from $S_a$ such that $\varvec{M}_{S_a}(\varvec{p}) = 1$ for all $\varvec{p}$ within $S_a$ and $\varvec{M}_{S_a}(\varvec{p}) = 0$ outside. Finally, $\varvec{M}_{S_a}$ is restricted by $\varvec{M}_{\text {R}}^h$ to generate the cavity label $\varvec{Y}_{\text {sim}}= \varvec{M}_{S_a} \odot \varvec{M}_{\text {R}}^h$, where $\odot$ represents the Hadamard product. Fig. 1 illustrates the process.

#### Simulating cavities filled with CSF

Brain RCs are typically filled with CSF. To generate a realistic CSF texture, we create a ventricle mask ${\varvec{M}_{\text {V}} : \varOmega \rightarrow \{ 0, 1 \}}$ from $\varvec{P}$, such that $\varvec{M}_{\text {V}}(\varvec{p}) = 1$ for all $\varvec{p}$ within the ventricles and $\varvec{M}_{\text {V}}(\varvec{p}) = 0$ outside. Intensity values within the ventricles are assumed to have a normal distribution [14] with a mean $\mu _{\text {CSF}}$ and standard deviation $\sigma _{\text {CSF}}$ calculated from voxel intensity values in $\{ \varvec{X}_{\text {pre}}(\varvec{p}) \mid \varvec{p}\in \varOmega \wedge \varvec{M}_{\text {V}}(\varvec{p}) = 1 \}$. A CSF-like image is then generated as $\varvec{X}_{\text {CSF}}(\varvec{p}) \sim {\mathcal {N}}(\mu _{\text {CSF}}, \sigma _{\text {CSF}}), \forall \varvec{p}\in \varOmega$.

We use $\varvec{Y}_{\text {sim}}$ to guide blending of $\varvec{X}_{\text {CSF}}$ and $\varvec{X}_{\text {pre}}$ as follows. A Gaussian filter is applied to $\varvec{Y}_{\text {sim}}$ to obtain a smooth alpha channel $\varvec{A}_\alpha : \varOmega \rightarrow [0, 1]$ defined as $\varvec{A}_\alpha = \varvec{Y}_{\text {sim}}* \varvec{G}_{{\mathcal {N}}}(\varvec{\sigma }),$ where $*$ is the convolution operator and $\varvec{G}_{{\mathcal {N}}}(\varvec{\sigma })$ is a 3D Gaussian kernel with standard deviations $\varvec{\sigma } = (\sigma _x, \sigma _y, \sigma _z)$. Then, $\varvec{X}_{\text {CSF}}$ and $\varvec{X}_{\text {pre}}$ are blended by the convex combination

5 $$\begin{aligned} \varvec{X}_{\text {sim}}= \varvec{A}_\alpha \odot \varvec{X}_{\text {CSF}} + (1 - \varvec{A}_\alpha ) \odot \varvec{X}_{\text {pre}}\end{aligned}$$

**Fig. 2:**
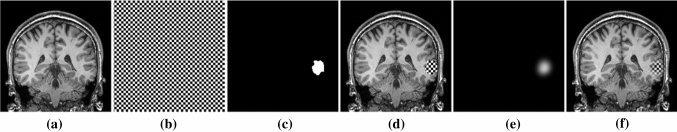
Simulation of resected image $\varvec{X}_{\text {sim}}$. We use a checkerboard for visualization. Two scalar-valued images $\varvec{X}_{\text {pre}}$ (**a**) and $\varvec{X}_2$ (**b**) are blended using $\varvec{Y}_{\text {sim}}$ (**c**) and $\sigma _i = {0}\,\mathrm{mm}$ to create an image with hard boundaries (**d**) and $\sigma _i = {5}\,\mathrm{mm}$ (**e**) for an image with soft boundaries (**f**), mimicking partial-volume effects

We use $\varvec{\sigma } > 0$ to mimic partial-volume effects at the cavity boundary. The blending process is illustrated in Fig. 2.

## Experiments and results

### Data

#### Public data for simulation

$T_{1}$w MRIs were collected from publicly available datasets Information eXtraction from Images (IXI), Alzheimer’s Disease (AD) Neuroimaging Initiative (ADNI) and Open Access Series of Imaging Studies (OASIS), for a total of 1813 images. They are used as control subjects in our self-supervised experiments (section “Simulation for domain adaptation and self-supervised learning”). Note that, although we use the term “control” to refer to subjects that have not undergone resective surgery, they may have other neurological conditions. For example, subjects in ADNI may suffer from AD.

#### Multicenter epilepsy data

We evaluate the generalizability of our approach to data from several institutions: *Milan* ($n = 20$), *Paris* ($n = 19$), *Strasbourg* ($n = 33$) and EPISURG ($n = 133$). We curated the EPISURG dataset from patients with refractory focal epilepsy who underwent resective surgery between 1990 and 2018 at the National Hospital for Neurology and Neurosurgery (NHNN), London, United Kingdom. All images in EPISURG were defaced using a predefined face mask in the Montreal Neurological Institute (MNI) space to preserve patient identity. In total, there were 430 patients with postoperative $T_{1}$w MRI, 268 of which had a corresponding preoperative MRI. EPISURG is available online and can be freely downloaded [21]. The same human rater (F.P.G.) annotated all images semi-automatically using 3D Slicer 4.10 [11].

#### Brain tumor datasets

The Brain Images of Tumors for Evaluation (BITE) dataset [19] consists of ultrasound and MRI of patients with brain tumors. We use 13 postoperative $T_{1}$w MRIs with gadolinium contrast enhancement ($T_{1}$wCE) to perform a qualitative assessment of our model’s generalization to images from a substantially different domain (contrast-enhanced images) and different pathology, where different surgical techniques may affect RC appearance.

#### Preprocessing

For all images, the brain was segmented using ROBEX [15]. They were resampled into the MNI space using sinc interpolation to preserve image quality. After resampling, all images had a 1-mm isotropic resolution and size $193 \times 229 \times 193$.

### Network architecture and implementation details

We used the PyTorch deep learning framework, training with automatic mixed precision (AMP) on two 32-GB TESLA V100 GPUs. We used Sacred [13] to configure, log and visualize experiments.

**Fig. 3:**
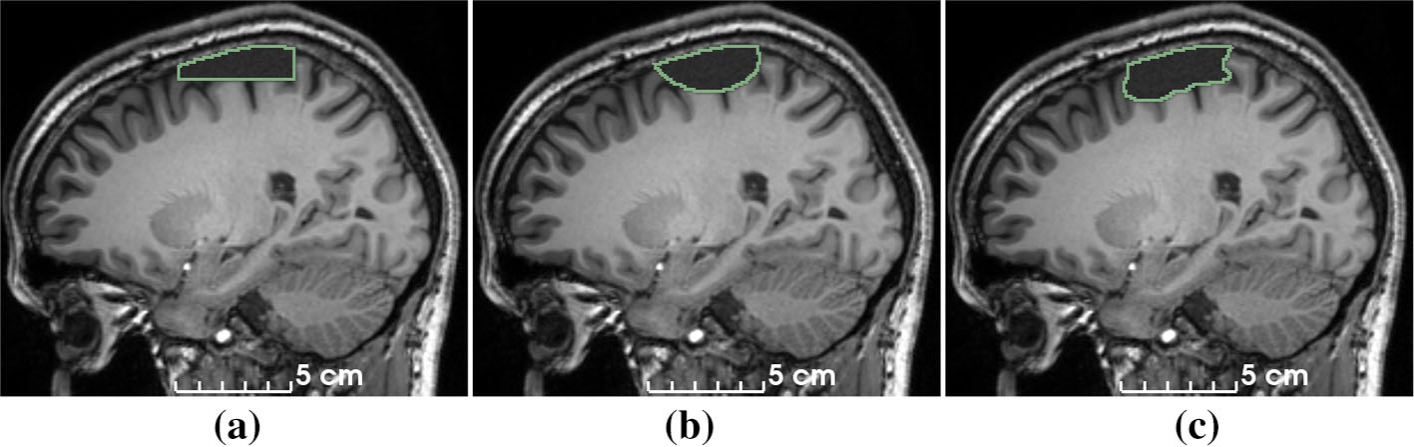
Simulation of RCs with increasing shape complexity (section “Resection simulation for self-supervised learning”): cuboid (**a**), ellipsoid (**b**) and ellipsoid perturbed with simplex noise (**c**)

We implemented a 3D U-Net [7] variant using two contractive and expansive blocks, upsampling with trilinear interpolation for the synthesis path and 1/4 of the filters for each convolutional layer. We used dilated convolutions, starting with a dilation factor of one, then increased or decreased in steps of one after each contractive or expansive block, respectively. Our architecture has the same receptive field (${88}\,\mathrm{mm}^3$) but $\approx 77 \times$ fewer parameters (246,156) than the original 3D U-Net, reducing overfitting and computational burden.

Convolutional layers were initialized using He’s method, and followed by batch normalization and nonlinear PReLU activation functions. We used adaptive moment estimation (AdamW) to adjust the learning rate, with weight decay of $10^{-2}$, and a learning scheduler that divides the learning rate by ten every 20 epochs. We optimized our network to minimize the mean soft Dice loss of each mini-batch. For training, a mini-batch size of ten images (five per GPU) was used. Self-supervised training took approximately 27 h. Fine-tuning on a small annotated dataset took approximately 7 h.

### Processing during training

We use TorchIO transforms to load, preprocess and augment our data during training [23]. Instead of preprocessing images with denoising or bias removal, we simulate different artifacts in the training instances so that our models are robust to artifacts. Our preprocessing and augmentation transforms are: (1) random simulation (RS) of resections (self-supervised training only), (2) histogram standardization, (3) Gaussian blurring or RS of anisotropic spacing, (4) RS of MRI ghosting, (5) spike and (6) motion artifacts, (7) RS of bias field inhomogeneity, (8) standardization of foreground to zero-mean and unit variance, (9) Gaussian noise, (10) RS of affine or free-form transformations, (11) random flip around the sagittal plane and (12) crop to a tight bounding box around the brain. We refer the reader to our GitHub repository for details.

### Experiments

Overlap measurements are reported as ‘median (interquartile range)’ DSC. No postprocessing is performed for evaluation, except thresholding at 0.5. We analyzed differences in model performance using a one-tailed Mann–Whitney *U* test (as DSCs were not normally distributed) with a significance threshold of $\alpha = 0.05$ and Bonferroni correction for *n* experiments: $\alpha _{\text {Bonf}} = \frac{\alpha }{n (n - 1)}$.

#### Self-supervised learning: training with simulated resections only

In our first experiment, we assess the relation between the resection simulation complexity and the segmentation performance of the model. We train our model with simulated resections on the publicly available dataset $D_{\text {pre}}= \{ \varvec{X}_{\text {preop}_i} \}_{i = 1}^{n_{\text {pre}}}$, where $n_{\text {pre}}= 1813$ (section “Data”). We use 90% of the images in $D_{\text {pre}}$ for the training set $D_{\text {pre,train}}$ and 10% for the validation set. At each training iteration, *b* images from $D_{\text {pre,train}}$ are loaded, resected, preprocessed and augmented to obtain a mini-batch of *b* training instances $\{ ( \varvec{X}_{\text {sim}_i}, \varvec{Y}_{\text {sim}_i}) \}_{i = 1}^{b}$. Note that the resection simulation is performed on the fly, which ensures that the network never sees the same resection during training. Models were trained for 60 epochs, using an initial learning rate of $10^{-3}$. We use the model weights from the epoch with the lowest mean validation loss obtained during training for evaluation. Models were tested on the 133 annotated images in EPISURG.

To investigate the effect of the simulated cavity shape on model performance, we modify $\phi _{\text {sim}}$ to generate cuboid-shaped (Fig. 3a) or ellipsoid-shaped (Fig. 3b) resections and compare with the baseline “noisy” ellipsoid (Fig. 3c). The cuboids and ellipsoid meshes are not perturbed using simplex noise, and cuboids are not rotated.

Best results were obtained by the baseline model [80.5 (18.7)], trained using ellipsoids perturbed with procedural noise. Models trained with cuboids and rotated ellipsoids performed significantly (57.9 (73.1), $p < 10^{-8}$) and marginally [79.0 (20.0), $p = 0.123$] worse.

#### Fine-tuning on small clinical datasets

We assessed the generalizability of our baseline model by fine-tuning it on small datasets from four institutions that may use different surgical approaches and acquisition protocols (including contrast enhancement and anisotropic spacing in *Strasbourg*) (section “Multicenter epilepsy data”). Additionally, we fine-tuned the model on 20 cases from EPISURG with the lowest DSC in section “Self-supervised learning: training with simulated resections only”.

For each dataset, we load the pretrained baseline model, initialize the optimizer with an initial learning rate of $5 \times 10^{-4}$, initialize the learning rate scheduler and fine-tune all layers simultaneously for 40 epochs using 5-fold cross-validation. We use model weights from the epoch with the lowest mean validation loss for evaluation. To minimize data leakage, we determined the above hyperparameters using the validation set of one fold in the *Milan* dataset.

**Fig. 4:**
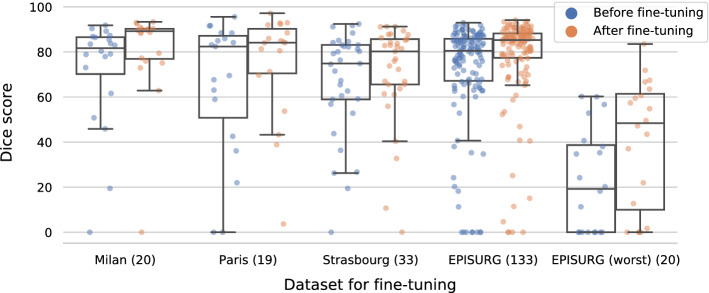
DSC without (blue) and with (orange) fine-tuning of the model training using self-supervision. Horizontal lines in the boxes represent the first, second (median) and third quartiles. *EPISURG (worst)* comprises the 20 cases from EPISURG with the lowest DSC in the experiment described in section “Self-supervised learning: training with simulated resections only”. Numbers in parentheses indicate subjects per dataset

We observed a consistent increase in DSC for all fine-tuned models, up to a maximum of 89.2 (13.3) for the *Milan* dataset. For comparison, inter-rater agreement between human annotators in our previous study was 84.0 (9.9) [22]. Quantitative evaluation is illustrated in Fig. 4.

#### Qualitative evaluation on brain tumor resection dataset

**Fig. 5:**
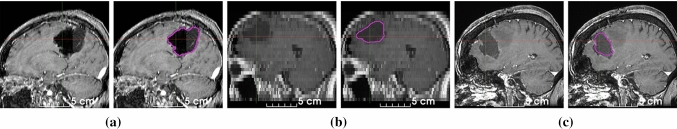
Qualitative results on postoperative brain tumor $T_{1}$wCE MRI. The model is robust to: air and CSF in the RC (**a**), anisotropic spacing (**b**), presence of edema (**c**) and a different modality than used for training (all). Note that these images are from a different institution, modality and pathology than the datasets used for quantitative evaluation. Manual annotations are not available

We used the BITE dataset [19] to evaluate the ability of our self-supervised model to segment RCs on images from a different institution, modality and pathology than the datasets used for quantitative evaluation. For postprocessing, all but the largest binary connected component were removed. The model successfully segmented the RC on 11/13 images, even though some contained challenging features (Fig. 5).

#### Qualitative evaluation on intraoperative image

**Fig. 6:**
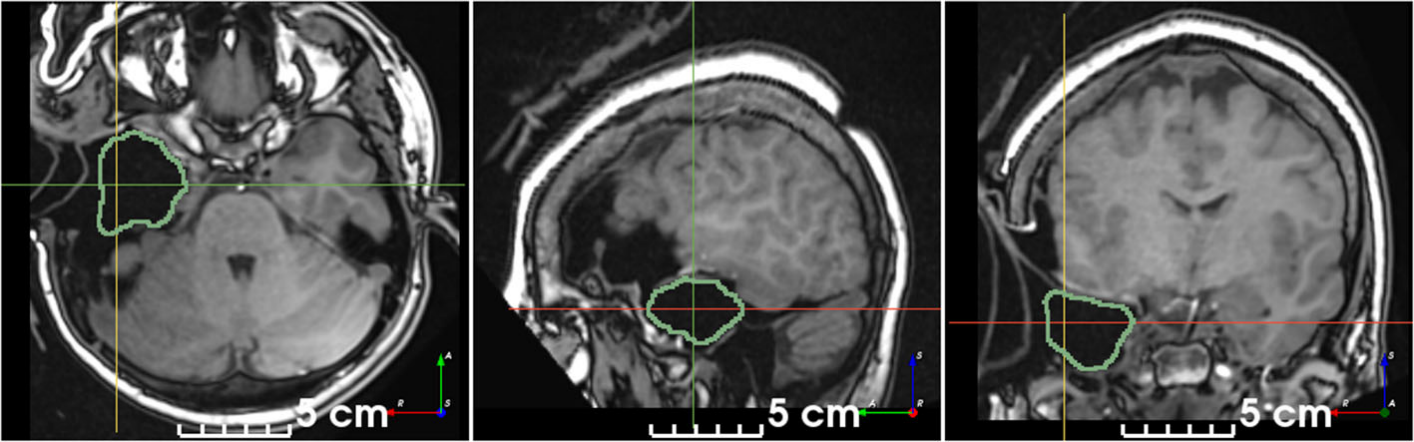
Qualitative result on an intraoperative MRI. The baseline model correctly discarded regions filled with air or CSF outside of the RC

We used our baseline model to segment the RC on one intraoperative MRI from our institution. Despite the large domain shift between the training dataset and the intraoperative image, which includes a retracted skin flap and a missing bone flap, the model was able to correctly estimate the RC, discarding similar regions filled with CSF or air (Fig. 6).

## Discussion and conclusion

We addressed the challenge of segmenting postoperative brain resection cavities from $T_{1}$w MRI without annotated data. We developed a self-supervised learning strategy to train without manually annotated data and a method to simulate RCs from preoperative MRI to generate training data. Our novel approach is conceptually simple, easy to implement and relies on clinical knowledge about postoperative phenomena. The resection simulation is computationally efficient ($< {1}\,\mathrm{s}$), so it can run during training as part of a data augmentation pipeline. It is implemented within the TorchIO framework [23] to leverage other data argumentation techniques during training, enabling our model to have a robust performance across MRI of variable quality.

Modeling a realistic cavity shape is important (section “Self-supervised learning: training with simulated resections only”). Our model generalizes well to clinical data from different institutions and pathologies, including epilepsy and glioma. Models may be easily fine-tuned using small annotated clinical datasets to improve performance. Moreover, our resection simulation and learning strategy may be extended to train with arbitrary modalities, or synthetic modalities generated from brain parcellations [1]. Therefore, our strategy can be adopted by institutions with a large amount of unlabeled data, while fine-tuning and testing on a smaller labeled dataset.

Poor segmentation performance is often due to very small cavities, where the cavity was not detected, and large brain shift or subdural edema, where regions were incorrectly segmented. The former issue may be overcome by training with a distribution of cavity volumes which oversamples small resections. The latter can be addressed by extending our method to simulate displacement with biomechanical models or nonlinear deformations of the brain [12].

We showed that our model correctly segmented an intraoperative image, respecting imaginary boundaries between brain and skull, suggesting a good inductive bias of human neuroanatomy. Qualitative results and execution time, which is in the order of milliseconds, suggest that our method could be used intraoperatively, for image guidance during resection or to improve registration with preoperative images by masking the cost function using the RC segmentation [2]. Segmenting the RC may also be used to study potential damage to white matter tracts postoperatively [27]. Our method could be easily adapted to simulate other lesions for self-supervised training, such as cerebral microbleeds [8], narrow and snake-shaped RCs typical of disconnective surgeries [20] or RCs with residual tumor [18].

As part of this work, we curated and released EPISURG, an MRI dataset with annotations from three independent raters. EPISURG could serve as a benchmark dataset for quantitative analysis of pre- and postoperative imaging of open resection for epilepsy treatment. To the best of our knowledge, this is the first open annotated database of postresection MRI for epilepsy patients.

## Data Availability

EPISURG can be freely downloaded from the UCL Research Data Repository [21].

## Footnotes

1 Note the volume of an ellipsoid with semiaxes (*a*, *b*, *c*) is $v = \frac{4}{3} \pi a b c$.

## Declarations

### Code availability

The code for resection simulation, training and inference is available at https://github.com/fepegar/resseg-ijcars. A tool to segment RCs using our best model (section “Self-supervised learning: training with simulated resections only”) can be installed from the Python Package Index (PyPI): pip install resseg.
